# Commentary: Improving ventricular assist device design. Much achieved with further innovation on the horizon

**DOI:** 10.1016/j.xjon.2020.07.002

**Published:** 2020-07-07

**Authors:** Nandan Kumar Mondal, Ravi Kiran Ghanta

**Affiliations:** a Division of Cardiothoracic Surgery, Michael E. DeBakey Department of Surgery, Baylor College of Medicine, Houston, Tex; b Division of Cardiothoracic Transplantation and Circulatory Support, Michael E. DeBakey Department of Surgery, Baylor College of Medicine, Houston, Tex

**Figure:**
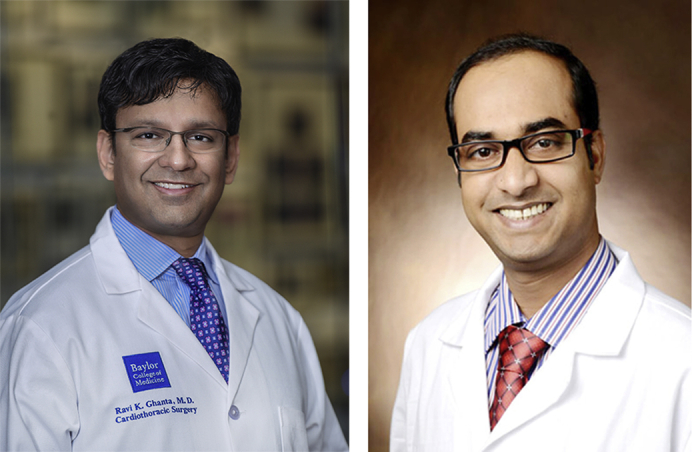
Ravi Kiran Ghanta, MD, FACS (*left*), and Nandan Kumar Mondal, MSc, MPhil, PhD (*right*)

**Central Message**

Miyamoto et al present a novel VAD design and demonstrate preliminary acute in vivo validation. They demonstrate pulse augmentation, easier weaning assessment, and biventricular possibilities.

See Article page 140.

Implantable ventricular assist devices (VADs) have been a “game-changer” in the management of end-stage heart failure as a bridge to heart transplantation, bridge to myocardial recovery, and as destination therapy. Since the initial pivotal REMATCH (Randomized Evaluation of Mechanical Assistance for the Treatment of Congestive Heart Failure) trial in 2001, VAD use has increased, technology has continued to advance, and biventricular options have been explored.^1^ Continuous-flow left VADs have become the dominant VADs used clinically due to their favorable size, energy efficiency, and durability over pulsatile LVADs.^2^^,^^3^ Challenges remain, specifically with bleeding, stroke, and pump thrombosis, requiring continued evolution and advancement of VAD technology.

In this article in *JTCVS Open*, Miyamoto and colleagues^4^ from the Cleveland Clinic present a novel VAD, which they call the “advanced VAD,” with preliminary validation in an acute in vivo study in calf models. Their VAD design has a rotor that moves axially to allow dynamic changes in aperture size based on pressure differential between the VAD inlet versus outlet. The investigators propose that this design enables performance over a wider range of pressures and use in a right VAD and left VAD setting. The authors presented this novel design in an in vitro benchtop model in 2016 and now have taken the next step to demonstrate in vivo feasibility.^5^ While this study is preliminary, the developed technology does address 2 interesting areas of VAD development. The first is the role of pulsatility. The physiologic implications of loss of pulsatility are unclear, with some hypothesizing that loss of pulsatility increases risk of gastrointestinal bleeding, pump thrombosis, vascular stiffening, organ fibrosis, and skeletal muscle wasting.^6^ Furthermore, in bridge-to-transplant patients, the return of pulsatility may also yield acute physiologic changes that may have implications in the post-transplant recovery period. The newest clinically approved VAD, the HeartMate 3 LVAD (Abbott, Chicago, Ill), creates an “artificial pulse” by software modulation of pump parameters.^7^ The “advanced VAD” by Miyamoto and colleagues provides pulse augmentation with an aortic pulse pressure of 15 mm Hg with flow dynamically varying from 0.2 to 6 L/min. This dynamic pulse augmentation is an interesting feature that can be further explored in chronic implantations. The second, important feature is related to pump weaning. For myocardial recovery, accurate assessment of underlying ventricular recovery is required to determine candidacy for VAD removal. The VAD design in this study prevents any regurgitant flow through the pump, which may be an important feature in clinical weaning assessment.

In addition to the authors' design changes, other important areas for future VAD development include the integration of computer modeling and biomaterials. Circulating blood is continuously experiencing nonphysiological elevated shear stresses and in direct contact with VAD material surfaces. The development of novel biomaterials may reduce thrombosis risk and further improve durability. A number of research groups are targeting the role of non-physiological high shear stress generated by VADs using computational fluid dynamics to identify “hotspots” of shear stress to further improve design.^8^ Furthermore, testing for hemolysis, platelet activation, and platelet dysfunctions is very important to understand how a new VAD can be responsible for blood damage in both in vivo animal feasibility experiments and human studies.^9^ Continued innovation in VAD design, as pursued by Miyamoto and colleagues, should be pursued to further improve clinical outcomes.
